# Applying differential network analysis to longitudinal gene expression in response to perturbations

**DOI:** 10.3389/fgene.2022.1026487

**Published:** 2022-10-17

**Authors:** Shuyue Xue, Lavida R.K. Rogers, Minzhang Zheng, Jin He, Carlo Piermarocchi, George I. Mias

**Affiliations:** ^1^ Department of Physics and Astronomy, Michigan State University, East Lansing, MI, United States; ^2^ Institute for Quantitative Health Science and Engineering, Michigan State University, East Lansing, MI, United States; ^3^ Department of Biological Sciences, University of the Virgin Islands, St Thomas, US Virgin Islands; ^4^ Department of Biochemistry and Molecular Biology, Michigan State University, East Lansing, MI, United States

**Keywords:** transcriptomics, RNA-seq, co-expression networks, gene expression, B cells, saliva

## Abstract

Differential Network (DN) analysis is a method that has long been used to interpret changes in gene expression data and provide biological insights. The method identifies the rewiring of gene networks in response to external perturbations. Our study applies the DN method to the analysis of RNA-sequencing (RNA-seq) time series datasets. We focus on expression changes: (i) in saliva of a human subject after pneumococcal vaccination (PPSV23) and (ii) in primary B cells treated *ex vivo* with a monoclonal antibody drug (Rituximab). The DN method enabled us to identify the activation of biological pathways consistent with the mechanisms of action of the PPSV23 vaccine and target pathways of Rituximab. The community detection algorithm on the DN revealed clusters of genes characterized by collective temporal behavior. All saliva and some B cell DN communities showed characteristic time signatures, outlining a chronological order in pathway activation in response to the perturbation. Moreover, we identified early and delayed responses within network modules in the saliva dataset and three temporal patterns in the B cell data.

## 1 Introduction

Network-based analysis, in particular, Differential Network (DN) analysis methods, have been very useful in analyzing the dynamics of gene expression under the effect of an external perturbation Ideker and Krogan (2012); Bandyopadhyay et al. (2010). DN analysis is a method based on the subtraction of one network from another and has been used in many genomics studies in the past decade Ha et al. (2015); Hsiao et al. (2016); Mitra et al. (2013). In a gene-gene correlation network (co-expression network), vertices represent genes while edges represent significant correlation of the expression of two genes Zhang and Horvath (2005). Typically, these co-expression networks are weighted by the strength and the sign of the correlation between two genes. The DN analysis method uses a pairwise cancellation of nodes and edges common to two networks that describe the expression data before and after a given perturbation. In doing so, the process leaves behind interaction variations that describe the network rewiring induced by the perturbation. For instance, in gene expression studies, DN analysis has been successfully implemented to separate gene expressions under specific drug responses from generic stress responses Cabusora et al. (2005). It also aided researchers in investigating dysfunctional gene regulatory networks in unhealthy states, providing insights into the genetic basis of diseases de la Fuente (2010). By focusing on the structural difference between two networks, the effectiveness of DN analysis has been demonstrated in identifying biological activities in different states.

When DN analysis is built from longitudinal gene expression data, there is also the opportunity to map the DN structure to key time-resolved features of the gene expression response to perturbations. For instance, identifying clusters of genes with common time activation and their temporal ordering. In the present study, we applied such a DN approach to RNA-sequencing (RNA-seq) time series datasets retrieved from two longitudinal RNA-seq experiments: (i) The first dataset (GSE108664) was generated from saliva samples from a healthy individual before and after the administration of the Pneumococcal Polysaccharide Vaccine (PPSV23) Mias et al. (2021). The primary goal of this study was to gain insights into the adaptive immune responses to PPSV23 through saliva profiling. Due to its convenience in processing relative to blood samples, saliva draws much interest for diagnostics as well as health monitoring applications. Saliva analysis can produce results in a timely manner, its collection is minimally invasive, and little training is required for saliva sampling, even for non-medically trained professionals. (ii) The second dataset (GSE100441) was generated from a time course experiment on primary B cells, where one set was treated with Rituximab and another used as an untreated control. Rituximab is known for its therapeutic use in targeting B cells Edwards et al. (2004) to treat cancers such as lymphomas and leukemias. This drug has a history of safe and effective usage since 1997 Bosch et al. (2014), and the World Health Organization (WHO) place Rituximab on their list of essential medicines World Health Organization (2021). Rituximab binds with CD20, expressed on pre-B and mature B cells, but not on stem cells, early pro-B or normal plasma cells Shaw et al. (2003). The binding causes perturbations to intracellular signaling and membrane structure Johnson and Glennie (2003), mediating the cell depletion. It is worthwhile mentioning that the B cell pathways of Rituximab activation have been experimentally validated Clynes et al. (2000); Jazirehi et al. (2003, 2005); Vega et al. (2005), and can be compared to the pathways identified by the DN method. Both the saliva and primary B cell experiments involve drug-treated samples (treatment sets) and untreated samples (control sets) monitored over time.

For both datasets, we started with building gene networks, one for each of the control and the treatment sets. We used gene-gene correlations between time series signals, over 24 h in saliva and 15 h in B cells, to evaluate pairwise gene connections. Graphically, the time series correlation networks built from the treatment sets summarized a system-wide pathway activation due to the perturbation, whereas the networks from the controls sets acted as the baseline. Within the DN analysis framework, we subtracted the baseline network from the one obtained using the treatment data, arriving at the final differential network.

The presence of modules, also known as communities, describes a topological property of networks Girvan and Newman (2002); Newman (2006); Fortunato (2010); Porter et al. (2009). One community is a group of densely connected nodes. In the context of a biological system, nodes in the same community are assumed to be close in biological functions Hartwell et al. (1999); Rives and Galitski (2003); Gulbahce and Lehmann (2008); Han (2008); Calderer and Kuijjer (2021). We exploited this property of the differential network to observe fine details of gene groups affected by the perturbation. That is, we employed one of the most established module detection algorithms, the Louvain method Blondel et al. (2008), to identify communities in our final differential network. We further explored communities by clustering heatmap and pathway enrichment analysis. The clustering heatmaps enabled us to characterize communities by their unique temporal patterns. Additionally, we identified early (and late) responding communities to perturbations and arrived at a sequential activation order for specifically the saliva DN communities. Lastly, we performed Reactome Croft et al. (2010) pathway enrichment analysis on individual communities and annotated the results with hub genes (based on DN centrality).

Our investigation extends applications of DN to gene expression time series that include perturbative activation. The two DN applications, and particularly the community-wide investigations provide further biological insight in gene expression changes in both Rituximab treatment, as well as pneumococcal vaccination. Specifically, each of the three investigations on DN communities provided unique perspectives on the biological response to perturbations: (i) Using our heatmap analyses, we found that each DN community can have its own temporal pattern and be used as a categorization of time-resolved gene activation. (ii) Using community enrichment, we determined the associations between the activated biological pathways and their gene clusters (communities). Combined with the community temporal patterns, our results provide a chronological order of pathway activations, and show how these may be obtained through a DN application. (iii) Lastly, our community hub analysis gave further insights into the biological functionality of individual genes in a community. These include, for example, the presence of the hub gene IL4R in one of the saliva DN communities, which suggests that the respective cluster of genes collectively activated T cells in response to the PPSV23 vaccine, and may explain a fever event in the experimental subject. Likewise, the presence of the hub gene PELI, known to be an oncogene in lymphomagenesis, in one of the B cell DN communities suggests that the entire community participates in the B cell response to Rituximab. Additional findings are summarized in the results below, and illustrate the utility of extending DN analyses to investigate time-resolved gene expression changes induced by drug and vaccine treatments.

## 2 Materials and methods

### 2.1 Data acquisition

Data for this investigation were obtained from Gene Expression Omnibus (GEO) for two time series studies using RNA-seq experiments, on Saliva (accession GSE108664) and Rituximab (GSE100441). Both sets of data are further described below. The raw RNA-seq data were mapped using Kallisto Bray et al. (2016), with bootstrap sample parameter, -b, was set to 100. GENCODE Harrow et al. (2012) v28 transcripts and genome built GRCh38.p12 were used for annotation. We used Sleuth Pimentel et al. (2017)(with DESeq Anders and Huber (2010) adjustment of Transcripts per Million) to compile results across timepoints.

The saliva dataset was obtained from our previous study of immune responses to the PPSV23 vaccine (GSE108664) Mias et al. (2021). In this study, hourly saliva samples were collected from a healthy individual over two 24 h periods and profiled with RNA-seq every hour. The first 24 h period provides a baseline RNA expression dataset, which we call *untreated* data. In the second 24 h period, the same individual was monitored after receiving the PPSV23 vaccine. Saliva samples were again collected hourly over 24 h and profiled by RNA-seq. This second step yielded the RNA expression dataset after the PPSV23 vaccination. We call these data the *treated* dataset. Both treated and untreated datasets have 24 time points of 84,647 possible expression signals using GENCODE annotation Harrow et al. (2012). We note that all data obtained were made publicly available by the original authors, Mias et al.Mias et al. (2021), as described therein (Michigan State University Institutional Review Board Protocol LEGACY15-071 [15–071]), and no additional institutional review board approvals were required for this investigation.

The perturbation in the primary B cell experiment was Rituximab, a monoclonal antibody drug used in the treatment of different types of lymphomas and leukemias. The experimental study (data from GSE100441) began by culturing in parallel primary B cells with and without Rituximab. During the 15 h of Rituximab treatment, the treated and untreated primary B cells were both sampled at the same six time points simultaneously and profiled by RNA-seq. The untreated group provided a baseline, which we call untreated data, whereas the treated experiment produced the treated dataset. Since this study included a replicated experiment, each of the first and second duplicates was processed to generate a separate network.

### 2.2 Data preprocessing

For quality control, we pre-processed the experimental data and filtered signals with multiple missing points right after importing the published data files. We coded all the data analysis in Python in this study. Using Python’s pandas package The Pandas development team (2020), McKinney (2010), we checked for missing values for each gene’s expression, removing duplicate records and eliminating genes with constant values across all the 24 time points for the saliva dataset (6 time points for the B cell datasets).

We replaced missing signals with zero and also set values less than 1 to 1. Genes with zero variance in their time series were excluded from our analysis. Moreover, we considered a gene signal as sparse and removed it if its time series had missing values for more than 1/8 of the time points. The same quality control procedure was used for both the saliva and primary B cell datasets.

### 2.3 Gene selection

After quality control, we further processed the data to pre-screen and identify a pool of candidate genes that showed response to the perturbations (vaccine in the saliva and Rituximab in the B cell). We selected genes that are highly expressed in both untreated and treated cases. Our goal with the differential networks was to identify genes that displayed notable changes. Hence the cutoffs were selected to exclude constant signals, and signals with moderate changes when comparing corresponding paired timepoints. For each of these genes, we calculated the time-averaged relative difference between treated and untreated normalized intensities, Δ_*TU*_:

$$\Delta_{TU}=\frac{1}{N}\underset{i}{\sum }\frac{T_{i}-U_{i}}{T_{i}+U_{i}}$$ (1)

where *T*_*i*_ is the expression value at time *i* in the treated dataset, *U*_*i*_ is the expression value at time *i* in the untreated dataset, and *N* is the total number of time points. This calculation yielded a Δ_*TU*_ distribution curve, from which we computed the lower and upper quartiles. As our goal was to identify time-resolved changes, genes were selected if their Δ_*TU*_s were within the bottom 25% or top 25% of the Δ_*TU*_ distribution respectively. The Python Pandas package was used for all the above computations The Pandas development team (2020), McKinney (2010).

### 2.4 Co-expression networks construction

After gene selection, we calculated their pairwise Pearson correlation coefficients and built the co-expression networks. Genes were represented as nodes and were joined by edges if there was a non-zero correlation between them. We used the co-expression coefficient as a weight for each edge. In the layout representation of the networks, the node-node distance reflects their correlation coefficients. Two genes are nearby if they have a high positive correlation. They are far apart when they have a low positive correlation or remote if negatively correlated. We used Python’s open source Networkx package Hagberg et al. (2008) for network visualization and calculation of the network metrics.

We constructed the network with edges in the 99.5% quantile of the correlation distribution, and excluding singletons. The one-sided quantile cutoff essentially selects for positive correlations and is consistent with our modularity-based community analysis discussed further below. For the saliva data, we built one treated and one untreated network. Since we have data from two repeated experiments for B-cells, we built two networks for the Rituximab treatment and two networks for the untreated control. Then, we took the intersections between the two networks corresponding to the repeats to obtain a single Rituximab-treated network and one single control network.

### 2.5 Differential networks construction

We defined the DN as the control network subtracted from the treated networks both for the saliva and B cell cases. In the subtraction, we remove an edge if that edge appears both in the treated and untreated networks. Edges appearing only in the treated network and absent in the untreated are kept in the differential network. Edges appearing only in the untreated network are not included. Isolated nodes left after this procedure are discarded. We analyzed the DN’s structure using modularity Girvan and Newman (2002); Newman (2006), as complex biological networks usually display a high degree of modularity Hartwell et al. (1999). Modularity is a measure to quantify relative edge densities from within the communities in comparison with those outsides. We utilized the Louvain community detection method Blondel et al. (2008), as implemented in a published Python package Bonald et al. (2020), a greedy algorithm for modularity maximization, to partition the entire DN into smaller clusters, also known as communities. The algorithm consists of two stages: first, individual nodes are joined into communities to achieve local maximum modularity; second, nodes within the same community are aggregated to form a new network where the node-assignment procedure is repeated until the modularity no longer increases. This graph clustering algorithm is not deterministic and can therefore result in slightly different partitions for the same graph. The partition yielded a few major components and many tiny communities of fewer than five genes, disconnected from the central islands. We pruned out these small communities from the DN. We found that the majority of our communities with a low number of genes yield no significant enrichment Reimand et al. (2019); Gene Set Enrichment Analysis (2022).

### 2.6 Community specific time-resolved analysis

In order to investigate the time-resolved response present within the communities, we applied clustering heatmaps to each of the DN communities. For genes in the same community, we first retrieved their treated and untreated expressions, then normalized each time series by subtracting individual time points from the time 0, followed by normalization with the Euclidean norm, for both expressions. We then took the difference between the normalized treated and untreated time series. Finally, we dendrogram-clustered these series (rows) with the complete-linkage method (Farthest Point Algorithm) Defays (1977); Hartigan (1985). The same procedure was repeated in all communities, and each delivered a clustering heatmap.

As the heatmaps rendered distinctive time-resolved responses in each community, we identified communities that responded quickly to perturbations and those that responded slowly. In particular, we characterized saliva communities by their peak times and arranged them in temporal order. We did not obtain an order for the B cell communities, as the B cell heatmaps did not show dominant peak times. However, we were still able to characterize B cell communities based on 3 distinguishable temporal pattern categories.

### 2.7 Pathway enrichment

We conducted Reactome Enrichment Analysis Croft et al. (2010) on each community to identify over-represented biological pathways within each community, using the Python package PyIOmica Domanskyi et al. (2020). As the majority of our communities with a low number of genes yield no significant enrichment, we focused on results for communities with 8 or more genes Reimand et al. (2019); Gene Set Enrichment Analysis (2022).

### 2.8 Community hub identification

Hubs are a typical feature in network topology. Visually, hubs represent highly connected nodes in a network. However, global connectivity differs from the regional structures. We isolated each community and identified localized hubs, only considering the communities rather than the global DN in our calculations. We adopted the standard Degree Centrality (DC) algorithm (which has been integrated with the Python networkx package) and identified the genes with the top five DC values as the community hub genes. We examined these hubs using functional annotations (such as pathway and memberships, including from GeneCards Suite Stelzer et al. (2016)) to evaluate if their biological properties could potentially elucidate the more general functionality of the module of their membership.

### 2.9 Results formatting and visualization

We stored the DN nodes and edges, communities, and pathway enrichment analyses into spreadsheets that are provided in the Online Data Files (ODFs) both for the saliva and B cell data. Using Mathematica Wolfram Research, Inc. (2021), we visualized the saliva and B cell DNs with their major connected components and communities.

## 3 Results

Our RNA-seq time series raw data were retrieved from the Gene Expression Omnibus (GEO) database under accessions GSE108664 and GSE100441 for the saliva and B cell experiments, respectively. The study of the immune response to the PPSV23 vaccine in saliva probed the expression of a potential 84,647 gene identifiers (GENCODE annotation Harrow et al. (2012)) at 24 time points Mias et al. (2021). The other study of drug activation by Rituximab in B cells provided a dataset for six time points. Since gene co-expression networks rely on correlations, our network analysis could be prone to spurious correlations, which we removed as described in the Methods section.

**FIGURE 1:**
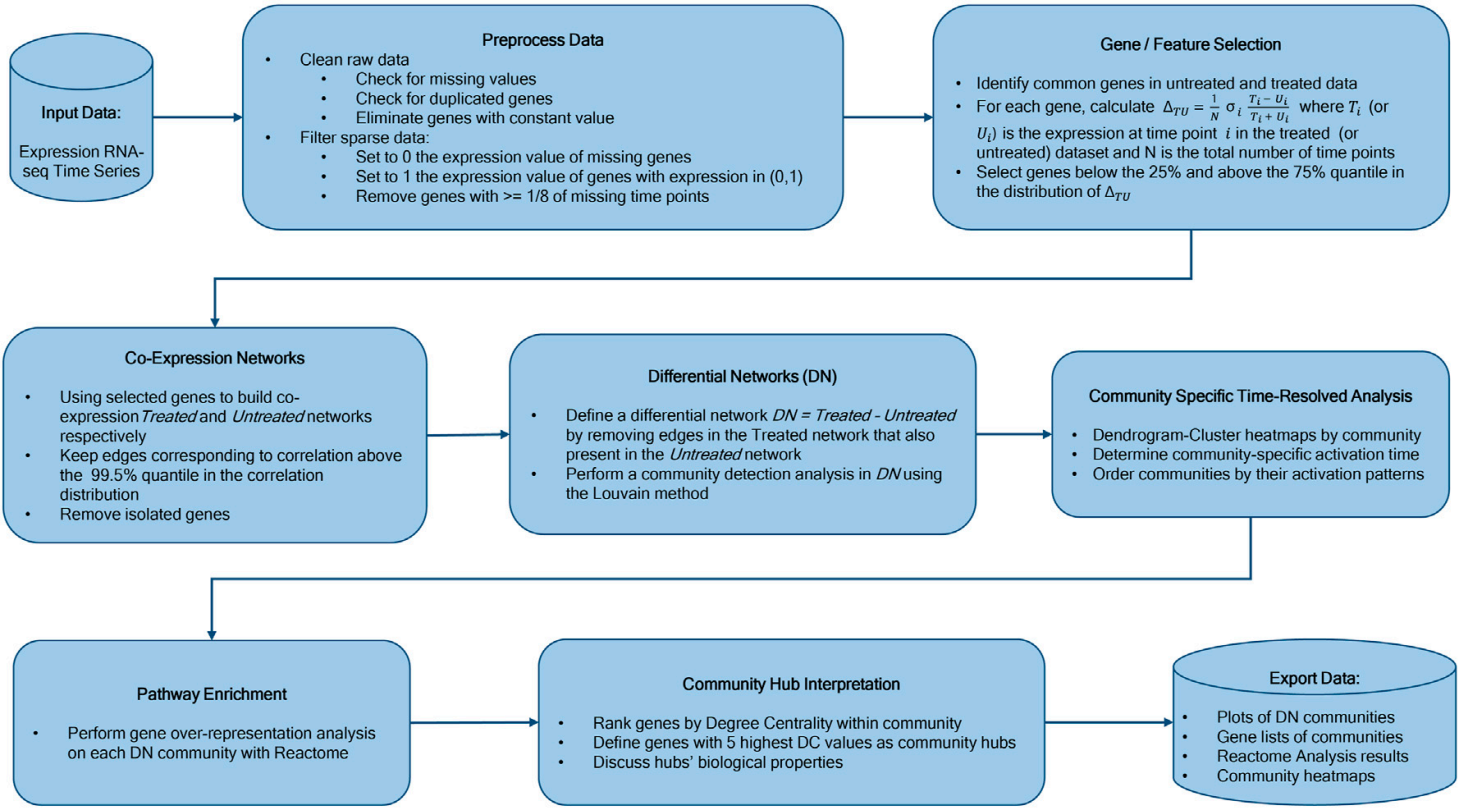
Workflow Overview. Our methodology starts with time course experimental data, followed by network construction, differential network determination, community detection, sequential ordering by activation pattern, pathway analyses of individual communities, community hub gene interpretation, and final results including analyses and temporal trend visualizations.

We constructed our saliva DN by subtracting the saliva network without vaccine from the network obtained using post-vaccine data. The B cell DN in response to Rituximab was generated in a similar manner. Next, we clustered the DNs into communities using the Louvain community detection method Blondel et al. (2008). We then conducted a Reactome Enrichment Analysis Croft et al. (2010) using PyIOmica Domanskyi et al. (2020), on each community to identify significant pathways and associated genes. We also visualized the heatmaps of relative gene expression as a function of time for each community. Finally, we plotted the DNs and their major individual communities. The workflow is summarized in Figure 1. See the Methods section for additional details.

### 3.1 Saliva DN

**FIGURE 2:**
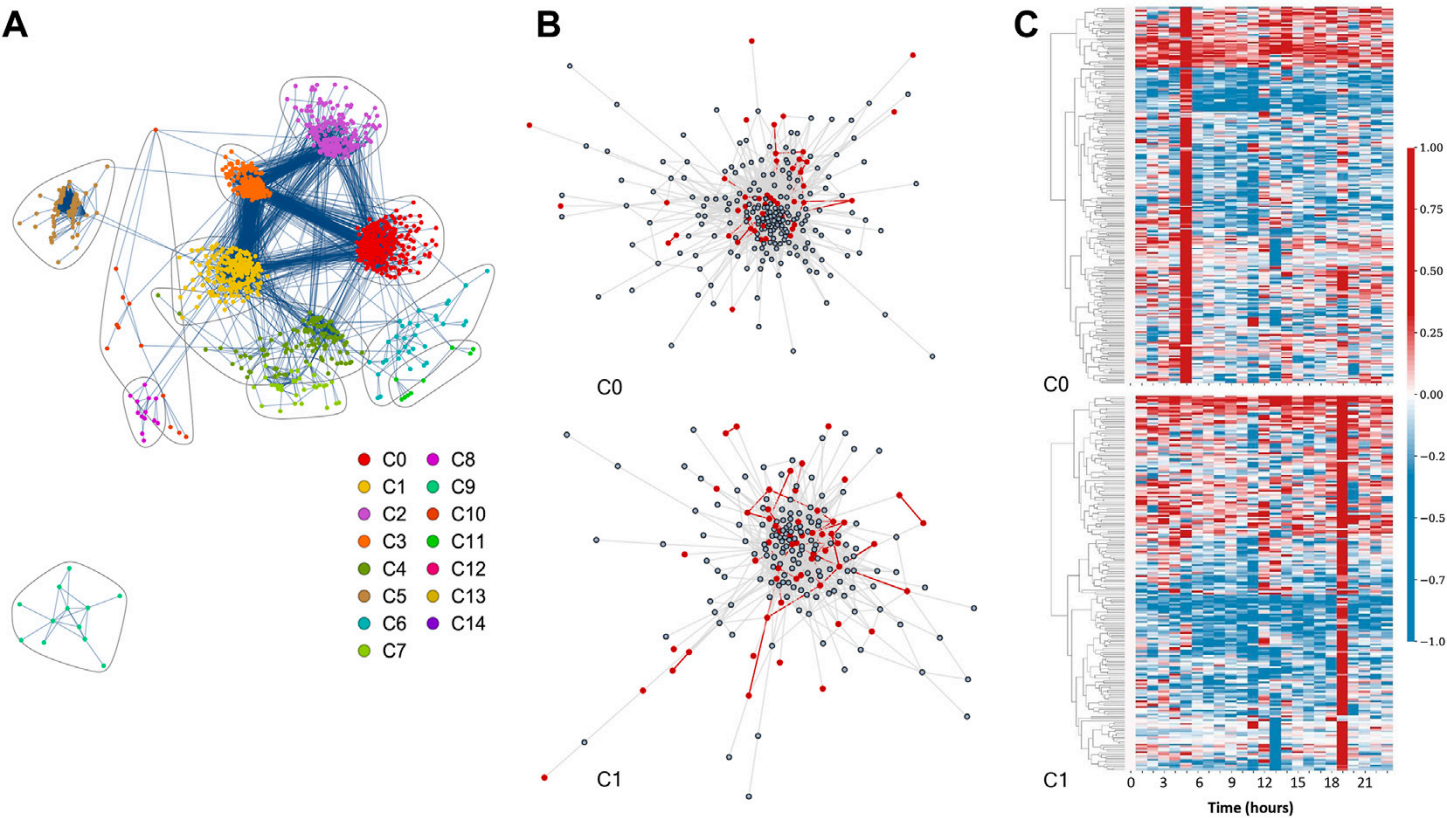
Differential network analysis for the saliva experiment. **(A)** Differential network with community structure found by the Louvain community detection method. **(B)** Isolated visualizations of C0 (top) and C1 (bottom) communities with red highlights indicating genes found in statistically significant Reactome pathways (FDR<0.05), and their corresponding edges in the network. **(C)** Heatmaps of C0 (top) and C1 (bottom) over 24 h. Columns represent time points while rows denote gene identifiers. The row data show the difference in each entry’s expression relative to time 0. The relative values were determined by subtracting the individual time points from time point 0 and then normalizing using a Euclidean norm, so that each row ranges from -1 (down-regulation) to 1 (up-regulation). For the dendrogram clustering we used the complete-linkage method (also known as the Farthest Point Algorithm) Defays (1977); Hartigan (1985).

Our saliva DN contains 1144 nodes (i.e., genes) and 13,775 edges. The Louvain algorithm identified 48 communities (modules) in total. 15 of the communities have a size of at least four nodes, while the remaining 33 are pairs or triplets. In the global saliva DN visualization, we excluded the communities with pairs or triplets, as none of them belonged to the three major connected components of the DN network. We also filtered the network to remove connected components with less than four genes. The global saliva DN is presented in Figure 2A, where communities are visualized using different colors and encircled in loops. Furthermore, community labels are based on their size (largest to smallest, with C0 being the largest community, and C14 the smallest).

### 3.2 Saliva communities temporal visualization

We further visualized each community’s change over time with heatmaps within the DN network. This is shown for C0 and C1 in Figure 2C. Here, each row denotes a gene, while each column corresponds to a time point (pre and post vaccination). The values plotted in the heatmaps are rescaled gene expression differences between the treated data and the control, and indicate the expression at the particular time point relative to the first time point of the experiment, with rows normalized using Euclidean norm. Red indicates up-regulated genes, blue down-regulated genes, and white indicates unchanged expression. The hierarchical clustering dendrograms revealed relationships among genes at each time point based on the similarity of the gene expressions. The prominent red columns show that genes are upregulated together at these time points. Note that the C0 heatmap has a pronounced peak at time point six, making C0 an early responding module, while C1 is a late responding cluster, with a pronounced peak at time point 19, as illustrated in Figure 2C.

Here we only show heatmaps for C0 and C1 as representative communities. However, we provide the other communities’ heatmaps and with their corresponding Reactome pathway analysis in the ODFs (folder “Results/SLV_results/network_plots”). Our saliva DN has a clear pattern of mostly discrete punctuated gene expression response times for each community. As these punctuated response times, save for one exception (both C0 and C11 show maximized response at t5), are specific to each community, they reflect the biological signatures for individual groups. Most of our saliva DN communities have only one punctuated activation time, although C5 in the saliva DN has 3 up-regulation events at time points 15, 20, and 22 that do not overlap with those of other communities. Between the communities, we observed strong temporally-specific relationships. Our heatmaps are suggestive of the presence of directional signaling between early-activation communities and subsequent groups, with a potential sequential activation pattern as follows: C6, C9, C8, C2, C0, and C1, C3, C4, C10, C5, C1, and finally C5. At time points from t6 to t10, t14, and from t16 to t18, no communities activated.

### 3.3 Pathway enrichment of saliva communities

In our pathway analysis, we queried individual communities to investigate how their highly co-expressed genes are functionally related. Our analysis is based on the Reactome pathway database Croft et al. (2010); Joshi-Tope et al. (2005); Matthews et al. (2009).

Statistically significant enrichment of pathways (with False Discovery Rate (FDR) < 0.05) was identified in six communities, C0, C1, C2, C4, C8 and C9. The majority of statistically significant Reactome pathways were related to response to stimulus, immune response, and inflammatory response. Among the six communities, C0 and C1 are the two largest communities. C0 comprises of 248 genes, colored in red in the global DN shown in Figure 2A, whereas C1 contains 198 genes, colored in yellow in the same panel. We display C0 and C1 in Figure 2B as representative communities. Genes that belong to the statistically significant biological pathways are highlighted in red in Figure 2B.

In the C0 community, the Reactome enrichment analysis identified 15 statistically significant pathways (FDR < 0.05): (i) three pathways for interferon signaling, (ii) three related to the immune system, (iii) four related to antigen presentation, (iv) one associated with ER-Phagosomes, (v) one lymphoid-related, and (vi) three pertaining to interleukin-12 signaling. In particular, the alpha, beta, and gamma signaling pathways all appear in the interferon signaling pathways. The immune system pathways include one cytokine signaling and one related to the adaptive immune system. Among the four antigen-related pathways, two are explicitly associated to the dependence of Class I MHC. The Endosomal/Vacuolar pathway implies the involvement of the Class I MHC and of the Antigen processing-Cross presentation. Lastly, interleukin-12 plays a crucial role in the coordination of innate and adaptive immunity Watford et al. (2003).

In the C1 community, the Reactome analysis identified 9 statistically significant pathways (FDR < 0.05). Two of these pathways are broadly related to the immune system and cytokine signaling. Another two pathways, the NGF-stimulated transcription and the FOXO-mediated transcription pathways, modulate cell survival, growth, and differentiation. In Table 1 we have listed all the results of the Reactome pathway enrichment analysis for C0 and C1 with FDR < 0.05.

**TABLE 1:** Reactome pathway enrichment analysis. Statistically significant pathways (FDR < 0.05) are summarized for saliva DN communities C0 and C1. In the full analysis, we omitted small communities with fewer than 8 genes Reimand et al. (2019); Gene Set Enrichment Analysis (2022), and 12 communities (C0 to C11) qualified for the pathway analysis.

Pathway Name	Entities FDR	Submitted entities found
*Saliva DN: C0*		
Antigen Presentation: Folding, assembly and peptide loading of class I MHC	1.2E-14	HLA-B, NAA15
Endosomal/Vacuolar pathway	1.2E-14	HLA-B
Interferon gamma signaling	1.2E-14	STAT1, IRF1, HLA-B, PTPN6
Class I MHC mediated antigen processing *and* presentation	1.2E-14	PSMD8, TLR1, CDH1, RPN1, GBF1, HLA-B, UBR4, CYBA, NAA15, ELOC, FBXO32, FBXO11
ER-Phagosome pathway	1.2E-14	PSMD8, TLR1, RPN1, HLA-B
Interferon alpha/beta signaling	1.2E-14	STAT1, IRF1, HLA-B, PTPN6
Interferon Signaling	1.2E-14	EIF4A1, STAT1, IRF1, HLA-B, PTPN6, ARIH1
Antigen processing-Cross presentation	1.2E-14	PSMD8, TLR1, RPN1, HLA-B, CYBA
Immunoregulatory interactions between a Lymphoid and a non-Lymphoid cell	1.2E-14	CDH1, CD81, HLA-B, FCGR2B
Cytokine Signaling in Immune system	1.1E-11	EIF4A1, STAT1, IRF1, HLA-B, PTPN6, ARIH1
Adaptive Immune System	4.1E-09	CD81, TCF25, RPN1, GBF1, HLA-B, UBR4, CYBA, PPP2R5D, FBXO32, FBXO11, ANKRD9, TLR1, PSMD8, CDH1, AKT2, PTPN6, ELOC, NAA15, FCGR2B, SIPA1, ARF5
Immune System	1.3E-05	CCDC71L, DDX3Y, EIF4A1, ASAH1, IL1RN, SERPINA1, TCF25, CD81, RPN1, RPLP0, UBR4, TNFAIP3, CSF2RA, PLD2, PSMD8, ANKRD9, CDH1, AKT2, OLR1, ELOC, ARIH1, SERPINB2, TNFSF14, GSTO1, STAT1, GBF1, HLA-B, CYBA, PPP2R5D, FBXO32, FBXO11, FGR, CEACAM3, CLEC4A, TLR1, IRF1, TCP1, TXNIP, PTPN6, CYSTM1, NAA15, FCGR2B, SIPA1, BIRC2, ARF5, TRIM56
Gene and protein expression by JAK-STAT signaling after Interleukin-12 stimulation	3.2E-03	SERPINB2, GSTO1, TCP1, RPLP0, ARF5
Interleukin-12 family signaling	4.6E-03	SERPINB2, GSTO1, STAT1, TCP1, RPLP0, ARF5
Interleukin-12 signaling	8.0E-03	SERPINB2, GSTO1, TCP1, RPLP0, ARF5
*Saliva DN: C1*		
Insulin-like Growth Factor-2 mRNA Binding Proteins (IGF2BPs/IMPs/VICKZs) bind RNA	2.7E-03	CD44
Nuclear Events (kinase and transcription factor activation)	1.8E-02	PPP2CB, TF, ID2, CHD4, FOS, DUSP6, DNM2
FOXO-mediated transcription of cell death genes	1.8E-02	BCL2L11, BCL6, NFYC
Signaling by NTRKs	2.4E-02	PPP2CB, RALA, TF, ID2, CLTA, FURIN, CHD4, FOS, DUSP6, DNM2
Signaling by NTRK1 (TRKA)	2.6E-02	PPP2CB, RALA, TF, ID2, CLTA, CHD4, FOS, DUSP6, DNM2
Immune System	2.9E-02	NAPA, RALA, CIITA, AHCYL1, RPN2, UNC93B1, JADE1, CLTA, BCL10, CFP, TANK, GNS, FCAR, STK10, PPP2CB, BCL2L11, TRIM29, ALOX5, NLRP3, FLNA, SIRPA, SLC12A6, IL6R, GBP4, RAP1GAP2, DDX17, CR1, WSB1, CISH, SH2D3C, KLHL21, FNDC3A, FOS, LILRB3, MTOR, DUSP6, VEGFA, DNM2, TF, ZNFX1, NASP, BCL6, MAN2B1, TACC2, CD300C, CALM1, CD44, LGMN
Cytokine Signaling in Immune system	3.6E-02	RALA, CIITA, CISH, RPN2, SH2D3C, FNDC3A, FOS, MTOR, DUSP6, VEGFA, PPP2CB, ZNFX1, BCL2L11, NASP, BCL6, TRIM29, ALOX5, FLNA, IL6R, GBP4, CD44
trans-Golgi Network Vesicle Budding	3.9E-02	NAPA, CPD, CLTA, GNS, CLINT1, DNM2
NGF-stimulated transcription	3.9E-02	TF, ID2, CHD4, FOS, DNM2

Of the communities we observed, the C0 community exhibits the strongest response to the stimulus and immune system, based on FDR $∼O\left(10^{-14}\right)$ . The complete pathway enrichment analysis for all communities in saliva is provided in the Online Data Files (ODFs), available on Zenodo, in the “Results/SLV_results/reactome_analysis” folder.

### 3.4 B cell DN

**FIGURE 3:**
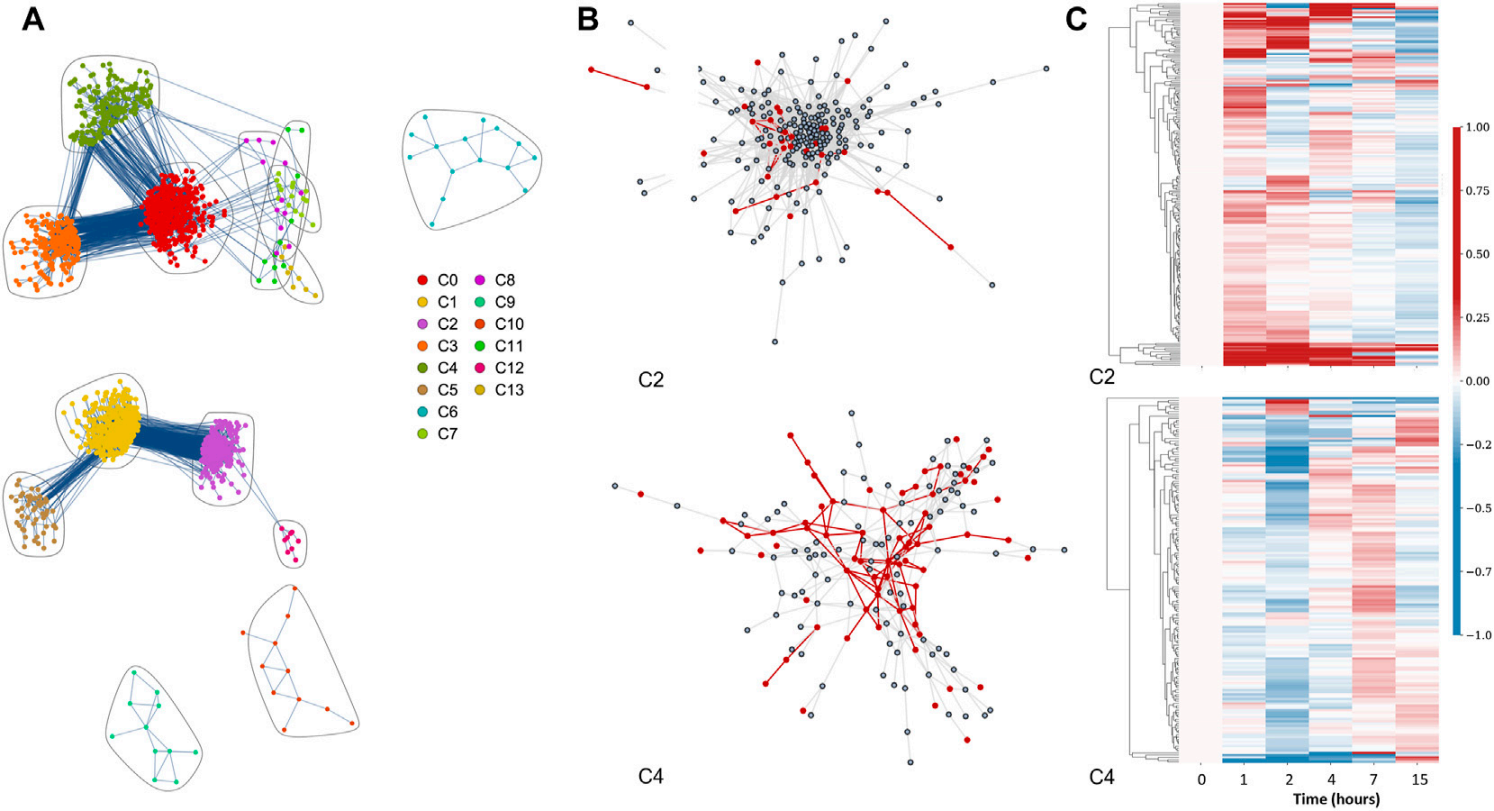
Differential network analysis for the B cell experiment. **(A)** Differential network with community structure found by the Louvain community detection method. **(B)** Isolated visualizations of C2 (top) and C4 (bottom) communities with red highlights indicating genes found in statistically significant Reactome pathways (FDR<0.05), and their corresponding edges in the network. **(C)** Heatmaps of C2 (top) and C4 (bottom) over 15 h (6 time points). Columns represent time points while rows denote genes. These row data demonstrate the difference in each entry’s expression relative to time 0. The relative values were determined by subtracting the individual time points from time point 0 and then normalizing using a Euclidean norm, so that each row ranges from -1 (down-regulation) to 1 (up-regulation). For the dendrogram clustering we used the complete-linkage method (Farthest Point Algorithm) Defays (1977); Hartigan (1985).

Our B cell DN consists of 1,759 nodes (genes) and 10,421 edges that we classified into 145 communities using the Louvain algorithm. Similar to the saliva DN, most of these communities are small clusters on small components. Due to its larger size relative to the saliva DN and larger number of communities, our cutoff for plotting was increased to 8 nodes both for community and component size. The global B cell DN is presented in Figure 3A, with five components and 14 communities. Here, we omitted the remaining 130 communities since they neither belong to any of the five main components, nor are they large enough for Reactome enrichment analysis. Like in the saliva DN, communities were ordered in descending size (largest to smallest, from C0 to C13 respectively), designated with different colors, and encircled by loops. Figure 3 has the same format of Figure 2. In this case, C2 and C4 are displayed in panel b, as magnified representations of the purple cluster and the green cluster, respectively, in panel a. Panel b’s magnified perspective provides details about the communities’ internal structures. In Figure 3B, for example, we observe that some of the genes highlighted in red form a clique.

### 3.5 Pathway enrichment of B cell communities

As for the saliva DN, we conducted a community-wise Reactome enrichment analysis for communities with at least 8 genes. 14 communities in the B cell DN were analyzed. This analysis found 9 communities with statistically significant pathway enrichment (FDR < 0.05.): C2, C4, C5, C6, C7, C9, C10, C12, and C13. Most of the pathways associated with genes in these communities centered around transcriptional regulation, protein metabolism, DNA binding ability, and signaling. Among its 111 statistically significant pathways, C4 was strongly enriched with genes in the FCERI-mediated NF-*κ*B activation pathway, the B cell receptor (BCR) signaling pathway, and the Fc epsilon receptor (FCERI) signaling pathway. These pathways and others relevant to Rituximab mechanism of action are listed in Table 2. The NF-*κ*B pathway activation by FCERI leads to the production of cytokines during mast cell activation, making it important in allergic inflammatory diseases Klemm et al. (2006). C4 also contained genes in the B cell receptor pathway, an important pathway related to B cells. The Fc epsilon gene is expressed on antigen-presenting cells, and its signaling occurs on the plasma membrane. A comprehensive list of statistically significant pathways can be found in the Online Data Files (ODFs) in the “Results/Bcell_results/reactome_analysis” folder.

**TABLE 2:** Reactome pathway enrichment analysis. Statistically significant pathways are summarized for primary B cell DN community C2 and C4. In the full analysis, we omitted small communities with fewer than 8 genes Reimand et al. (2019); Gene Set Enrichment Analysis (2022), and 14 communities (C0 to C13) qualified for the pathway analysis.

Pathway Name	Entities FDR	Submitted entities found
*B cell DN: C2*		
Peptide chain elongation	1.3E-06	EEF1A1, RPL4, RPL7A, RPL27A, RPS6, RPL36, RPL14, RPS20, RPL15, FAU, UBA52, RPL28
Response of EIF2AK4 (GCN2) to amino acid deficiency	1.3E-06	RPL4, RPL7A, RPL27A, RPS6, RPL36, RPL14, RPS20, FAU, RPL15, UBA52, RPL28, ATF3
Eukaryotic Translation Elongation	1.6E-06	EEF1A1, RPL4, RPL7A, RPL27A, RPS6, RPL36, RPL14, RPS20, RPL15, FAU, UBA52, RPL28
GTP hydrolysis and joining of the 60S ribosomal subunit	5.9E-06	RPL4, EIF4A1, RPL7A, RPL27A, RPS6, RPL36, RPL14, RPS20, RPL15, FAU, UBA52, RPL28
L13a-mediated translational silencing of Ceruloplasmin expression	5.9E-06	RPL4, EIF4A1, RPL7A, RPL27A, RPS6, RPL36, RPL14, RPS20, RPL15, FAU, UBA52, RPL28
Nonsense Mediated Decay (NMD) independent of the Exon Junction Complex (EJC)	5.9E-06	RPL4, RPL7A, RPL27A, RPS6, RPL36, RPL14, RPS20, RPL15, FAU, UBA52, RPL28
Formation of a pool of free 40S subunits	7.7E-06	RPL4, RPL7A, RPL27A, RPS6, RPL36, RPL14, RPS20, RPL15, FAU, UBA52, RPL28
Eukaryotic Translation Termination	7.7E-06	RPL4, RPL7A, RPL27A, RPS6, RPL36, RPL14, RPS20, RPL15, FAU, UBA52, RPL28
Cap-dependent Translation Initiation	9.2E-06	RPL4, EIF4A1, RPL7A, RPL27A, RPS6, RPL36, RPL14, RPS20, RPL15, FAU, UBA52, RPL28
Eukaryotic Translation Initiation	9.2E-06	RPL4, EIF4A1, RPL7A, RPL27A, RPS6, RPL36, RPL14, RPS20, RPL15, FAU, UBA52, RPL28
*B cell DN: C4*		
Metabolism of RNA	1.4E-02	SF3B4, MT-ND6, NUP205, UTP3, POP1, DDX23, CSTF2, PHAX, PLRG1, DIEXF, ZFP36L1, FTSJ3, CHERP, PSMD8, EFTUD2, PSMD9, PSMC4, PSME3, NUP35, SKIV2L2
Mitotic Anaphase	1.4E-02	PSMD8, PSMD9, NUP205, CCNB1, SPAST, PSMC4, PSME3, NUP35, SMC1A, EMD, KPNB1
Mitotic Metaphase and Anaphase	1.4E-02	PSMD8, PSMD9, NUP205, CCNB1, SPAST, PSMC4, PSME3, NUP35, SMC1A, EMD, KPNB1
FCERI mediated NF-kB activation	1.4E-02	IGLV2-11, PSMD8, PSMD9, IGKV2-29, IGKV1-16, PSMC4, PSME3, IGKV4-1
Signaling by the B Cell Receptor (BCR)	1.4E -02	IGLV2-11, PSMD8, PSMD9, IGKV2-29, IGKV1-16, PSMC4, PSME3, IGKV4-1, PIK3AP1
Fc epsilon receptor (FCERI) signaling	1.4E-02	IGLV2-11, PSMD8, PSMD9, IGKV2-29, IGKV1-16, PSMC4, PSME3, IGKV4-1
Host Interactions of HIV factors	1.4E-02	PSMD8, PSMD9, NUP205, PSMC4, PSME3, NUP35, KPNB1
G1/S Transition	1.4E-02	PSMD8, PSMD9, CCNB1, MCM7, PSMC4, PSME3, KPNB1
ABC-family proteins mediated transport	1.4E-02	PSMD8, PSMD9, PSMC4, PSME3, CSTF2, EIF2S1
Assembly of the pre-replicative complex	1.4E-02	PSMD8, PSMD9, MCM7, PSMC4, PSME3

In summary, C4 contains the highest number of responsive pathways relevant to the B cell response to Rituximab. As our representative communities, we display the C2 and C4 in Figure 3B, our two largest among the 9 communities with statistically significant pathways. Our top 10 pathways based on FDR from the Reactome enrichment analysis for C2 and C4 are listed in Table 2.

### 3.6 B cell communities temporal visualization

The heatmaps for the temporal behavior for the C2 and C4 communities of the B cell data are shown in Figure 3C. The formatting of the heatmaps is the same as that of the saliva heatmaps; all values in the heatmaps refer to gene expression relative to time 0 in the treated dataset. The C4’s blue column at time point 2 and the less prominent blue column for C2 at time point 15 identify patterns of down-regulation in the two communities. While C2 shows a trend of initial up-regulation followed by a gradual diffusion, C4 exhibits an initial down-regulation, followed by later up-regulation.

Though C2 and C4 are our representative communities, we carried out heatmap visualization for all 9 communities that demonstrated statistically significant pathway enrichment. These heatmaps are available to view in the ODFs in the “Results/Bcell_results/network_plots/heatmaps” folder. Overall, in the B cell community heatmaps, we recognized three types of time patterns in terms of collective behavior within an individual community. In the first pattern group, the majority of genes started with a moderate degree of down-regulation. By 7 h, most instead displayed slight or moderate up-regulation. However, each of these timepoints contained a minority of genes with a small level of fluctuation, with the size of the deviating group differing in each heatmap. The second observed time pattern operated in reverse, with most genes beginning upregulated and shifting towards downregulation by the 15-h mark. Finally, a third group remained consistent in its behavior, with genes trending one way or remaining unchanged across the entire time period.

### 3.7 Community hubs

We examined the community hub genes for both saliva and B cell DNs, and reported the degree centrality in their respective Results tables in the ODFs with sheet name “Degree Centrality”. When two DC values are the same, the genes are tied in rankings in our consideration as community hubs.

### 3.8 Biological considerations

In our results, a number of expected pathways emerged. These included pathways associated with antigen presentation and processing, Class I MHC mediated antigen processing and presentation, and ER-phagocytosis, and pathways governing the immunoregulation of interactions between Lymphoid and non-Lymphoid cells Joshi-Tope et al. (2005). Further results indicative of the participation of immune cells, included the CLEC inflammasome pathway in C4. This pathway is associated with enabling host immune system to mount a fungal/bacterial defense using T-Helper 17 cells (TH17) Gringhuis et al. (2012); Cheng et al. (2011). Interferon signaling, cytokine signaling, immune/adaptive immune, and interleukin stimulation and signaling are all part of a generalized immune response Abbas et al. (2018). We found these more general pathways in the pathway enrichment analysis of C0, C1, C2, C9, and C10. Interferon signaling is crucial in antiviral defense, cell regulation and growth, and immune response modulation Bonjardim et al. (2009). Our Reactome pathway analysis results are consistent with the results of our saliva multi-omics study Mias et al. (2021), which observed that vaccination activates various immune response and regulation pathways, which are also identified in our present results, including ER-Phagosome pathway, Interferon alpha/beta and gamma signaling, cytokine signaling, and MHC antigen presentation.

From our community hub gene analysis, a few hub genes are suggestive of community functionality. Notably, the community C2 hub gene, BIRC2, regulates NF-*κ*B signaling as well as inflammatory signaling and immunity Liu-Mares et al. (2007); Consortium (2021). For one of the C2 hubs, URGCP, previous findings indicate that its upregulation and downregulation are significantly involved in the molecular mechanisms of non-small cell lung cancer Cai et al. (2015); Consortium (2021). Accordingly, the presence of the URGCP is consistent with our vaccine targeting the respiratory system. As for community C3, the IL4R gene encodes interleukin four and interleukin 13 to regulate IgE production, which further activates the JAK/STAT pathway Bhattacharjee et al. (2013). This pathway orchestrates cytokine receptors, modulates T helper cell polarization, and also mediates human monocytes/macrophages Bhattacharjee et al. (2013); Seif et al. (2017). Lastly, in community C5, the EBF1 is known as a leading transcription factor of B-cell specification Yang et al. (2016). In summary, IL4R and EBF1 became the most connected gene in C3 and C5, respectively, which implies that these two communities are each centered around T cells (C3) and B cells (C5). Moreover, given that the experimental subject had a post-vaccination fever at hour 11 Mias et al. (2021), at which our C3 heatmap coincidentally peaks (see figure in ODFs), the hub gene IL4R in C3 appears to relate the fever event with T cell responses.

Regarding our primary B cell results, previous work Seyfizadeh et al. (2016) has established both the biological pathways and the mechanisms of action associated with Rituximab. These previous studies have demonstrated Rituximab’s ability to cause antibody-dependent and complement-dependent cellular cytotoxicity, growth inhibition and apoptosis, and regulation of the cell cycle. We also expected to observe Rituximab regulations of the B cell receptor (BCR) based on prior research. Notably, our findings included the enrichment of the nuclear factor *κ*B (NF-*κ*B) pathways. According to Jazirehi et al. (2005) Jazirehi et al. (2005) and Bonavida (2007), Jazirehi and Bonavida (2005), treating Non-Hodgkin’s lymphoma (NHL) B cell lines with Rituximab inhibits NF-*κ*B’s signaling pathways by up-regulating RKIP and Raf-1 kinase inhibitors. RKIP has been found to antagonize signal transduction pathways that mediate the NF-*κ*B activation Yeung et al. (2001).

Following NF-*κ*B’s down-regulation due to RKIP’s up-regulation, the Bcl-xl expression is also down-regulated. As a result, tumor cells become more chemosensitive. Rituximab also decreased the activity of NF-*κ*B-inducing kinase, IkB kinase, and IkB-a phosphorylation. Finally, the introduction of Rituximab also decreased the activity of the IKK kinase and NF-*κ*B binding to DNA from 3 to 6 h after treatment Jazirehi et al. (2005).

Among the more general enriched pathways observed are signaling pathways that play a role in the molecular mechanisms of chemosensitization, which are also impacted by Rituximab. In line with those effects, we anticipate impacts in the MAPK signaling pathway, the interleukin cytokine regulatory loop, and the Bcl-2 expression. Concerning the expression of genes involved in the healing process, research has uncovered Rituximab’s role in affecting pathways associated with immunoglobulin production, chemotaxis, immune response, cell development, and wound healing. Rituximab can also increase existing drug-induced apoptosis Seyfizadeh et al. (2016).

In our community of C4, for example, our Reactome analysis found 5 NF-*κ*B related pathways with FDR < 0.05. Of these five pathways, one is shown in Table 1; the remaining are displayed in the comprehensive table in the ODFs (“Results/Bcell_results/reactome_analysis” folder). Alongside these NF-*κ*B pathways in C4 is the BCR pathway. Our results suggest that the C4 community response is highly relevant because of the activation of both NF-*κ*B and BCR pathways.

Our C2 community appears to be involved with the metabolism of proteins and cellular responses to external stimuli. Rituximab targets the CD20 B cell transmembrane protein that is involved in B-cell development, activation and proliferation Seyfizadeh et al. (2016). The C2 community captures cell development pathways included in our expectations of more generalized responses.

We also observed relevant responses in other communities. For example, the C8 community showed activity in the RAF/MAP kinase cascade pathway. In a similar fashion, C10 demonstrated CD22 mediated BCR regulation, classical antibody-mediated complement activation, FCGR activation, antigen activation of the BCR, and initial complement triggering, *etc.* The pathways that emerged in our results are thus consistent and highly overlap with established pathways from previous studies.

Hub genes most pertinent to B cell/lymphocytes included PELI1 in community C5, PRDM2, MALAT1, and SND1 in C2. Other high centrality genes with similar relevance included MAPK8 in C6 and AFF3 in C1. Among these, PELI1 turned out to be closely associated with antitumor immunity in B cells, which is the therapeutic goal of the Rituxmab treatment. A previous study Park et al. (2014) showed that prolonged expression of PELI1 causes B cell hyperactivation, which, in turn, promotes various lymphoid malignancies. To a lesser extent, an increased expression of PELI1 can induce BCL6, an oncoprotein known for advancing lymphomagenesis, for example, B-acute lymphoblastic leukemia and chronic myeloid leukemia. This pathway has been recognized as a potential therapeutic target for treating B cell lymphoma. Out of the C2 hub genes, PRDM2 is a tumor suppressor Steele-Perkins et al. (2001), whereas the upregulation of MALAT1 is linked to tumor cell proliferation and metastasis, such as leukemia Huang et al. (2017). The protein encoded by gene SND1 is known to interact with Epstein-Barr virus nuclear antigen 2 (EBNA 2), which is essential for B-lymphocyte transformation Tong et al. (1995). As an oncogene Blanco et al. (2011), SND1 has attracted clinical investigation as a cancer treatment candidate due to its association with cell proliferation, and malignant transformation Ochoa et al. (2018). The literature also has reported that MAPK8, which we found in community C6, mediates starvation-induced BCL2 phosphorylation Wei et al. (2008), a sign of cell apoptosis, and AFF3, found in community C1, serves a role in lymphoid maturation and oncogenesis Hiwatari et al. (2003). The fact that these genes appear as hubs in the Rituximab’s DN is consistent with their known important roles in B-cell malignancies and merits further investigation.

## 4 Discussion

Our goal was to use a DN approach to identify the activation of biological processes caused by a perturbation in saliva and primary B cells. This study applied DN analysis, community identification and Reactome pathway analysis of the DN communities, and identified communities with highly statistically significant enrichment. In this study we implemented a modularity-based community detection, that works with positive correlations. This is a limitation of the modularity approach to the DN that may be addressed using different community detection algorithms and merits followup investigations. We analyzed the DNs of two gene expression datasets where a perturbation was applied: (i) Saliva dataset (PPSV23 vaccination as perturbation; 24 time points), (ii) Primary B-Cells dataset (*ex-vivo* Rituximab drug treatment as perturbation; six time points). In summary, our results from the saliva DN revealed pathway activation in immunological and inflammatory responses. In the B cell DN, statistically significant pathways were activated in the regulation of transcription, immune cell survival, activation and differentiation, and inflammatory response.

*Streptococcus Pneumoniae*’s virulence and associated host immunity have been extensively studied Brooks and Mias (2018). The PPSV23 is an inactivated vaccine that uses purified capsular polysaccharides, and is typically administered to older adults (65+) and susceptible younger individuals US Food and Drug Administration (2014); Butler et al. (1993); Smit et al. (1977); Pletz et al. (2008). In our analysis, we focused on the vaccine’s potential pathways of action. Our initial saliva investigation in PPSV23 established that an immune response to the vaccination can be detected utilizing non-invasive saliva monitoring at the molecular level Mias et al. (2021). Since aggregate saliva was sampled, we expected that multiple immune cells contained therein are involved in the observed patterns and associated immune responses. Based on our previous findings and general vaccine responses, we anticipated the activation of pathways involved with antigen presentation and processing, regulation of IgM and B/T cells, Lymphoid cells, MHC molecules, and phagocytosis. We also expected the activation of pathways of general immune response to stimuli or inflammation.

Communities aid in defining the genes’ collective behavior, and observing the collective behavior of communities in the entire network can clarify relative trends between these collective behaviors. The generated heatmaps for each community depicted gene regulation for individual time points, and also displayed trends over time within the identified communities. The trends we observed in our saliva data were consistent with a time-dependent regulation. The results suggest a sequence of communities activations (up- and down-regulation) at individual timepoints, indicative of sequential immune system responses due to the PPSV23 vaccination. In the primary B cell, data were less clear, as fewer time points were monitored, and also the network was more densely connected. The B-cell heatmaps still indicate overall trends associated with Rituximab activation (both up- and down-regulation) within the first 7 h of the treatment. Our future work will focus on the possibility of establishing a causal chained signaling response, and associated pathways across these communities.

Our analysis showed the applicability of a DN approach in evaluating time course RNA-seq data. Specifically, the DN method results in the saliva experiment data were consistent with our previous work on profiling PPSV23 vaccination responses Mias et al. (2021). For the primary B cell responses to Rituximab, the DN has found the same signaling pathway as numerous prior experimental results, thus helping with our validation from a computational perspective. The DN approach complements prior studies by offering a systems-level network perspective of aggregate temporal changes due to drug activation. In future work we plan to address the identification of sequential activation of network communities, as well determining directionality/causality in such activations.

## Data Availability

Publicly available datasets were analyzed in this study. These data can be found here: Gene Expression Omnibus (GEO; https://www.ncbi.nlm.nih.gov/geo/), accessions: (i) GSE108664 for the saliva mRNA-sequencing, and (ii) GSE100441 for the Rituximab Treatment in Primary B Cells mRNA-sequencing. Mapped RNA-seq data have been deposited on Zenodo and are publicly available at: http://dx.doi.org/10.5281/zenodo.7007320. Results and code files have also been deposited on Github (https://github.com/gmiaslab/DifferentialNetworks) and released on Zenodo. These files are referred to as Online Data Files (ODFs) in the manuscript. DOI: http://dx.doi.org/10.5281/zenodo.7007320.
